# Laser Interstitial Thermal Therapy (LITT) for Neurosurgical Procedures: Facts You Need to Know About the Anesthetic Management of LITT Procedures

**DOI:** 10.7759/cureus.53920

**Published:** 2024-02-09

**Authors:** Lakshmi N Kurnutala

**Affiliations:** 1 Anesthesiology and Perioperative Medicine, University of Mississippi Medical Center, Jackson, USA

**Keywords:** litt, mri, radiation necrosis, stereotaxic, epilepsy, brain tumors

## Abstract

The field of medicine is constantly advancing to improve patient care. As physicians, we must improve our knowledge by listening, reading, and practicing evidence-based medicine. Laser treatment has evolved over the years in many surgical specialties. Laser interstitial thermal therapy (LITT), also known as stereotactic laser ablation (SLA), was developed in neurosurgical procedures to treat recurrent or metastatic brain tumors, radiation necrosis, and epilepsy lesions. LITT procedures are advantageous in providing better patient outcomes, decreased hospital length of stay, and reduced total hospital cost. These procedures are performed as a multi-disciplinary approach; this article discusses the different types of LITT systems, indications, contraindications, types of anesthesia, perioperative anesthetic management, safety precautions, complications, recovery during and after LITT procedures, and the future of LITT procedures.

## Introduction and background

Laser interstitial thermal therapy (LITT) is used in multiple neurosurgical brain and spine procedures. Recently, many medical centers across the globe started performing these procedures to improve the care and outcomes of patients with brain and spine tumors. LITT procedures are performed using a team approach that involves neurosurgery, neurology, radiation oncology, radiology, anesthesiology, and ancillary staff [1,2]. The coordination between the multiple specialties starts with identifying the right patient for the LITT procedure. LITT procedures are expected to improve patient safety, lower patient morbidity, shorten hospital stays (1-2 days), reduce hospital costs, and increase the potential for long-term survival in primary or secondary metastatic tumors of the brain and spine. With the increase in the number of new procedures using LITT, physicians and medical staff need to familiarize themselves with various considerations related to these procedures. This article discusses the top ten facts physicians should know about anesthesia for LITT procedures.

## Review

History and introduction of LITT

Sugiyama et al. first performed and described the stereotactic LITT procedures using an Nd:YAG (neodymium-doped yttrium aluminum garnet) laser [3]. LITT procedures, also known as stereotactic laser ablation (SLA), are minimally invasive procedures that use a localized laser to destroy the unhealthy tissue of the brain. LITT procedures are performed for deep-seated brain tumors, recurrent brain tumors, metastatic brain tumors, radiation necrosis, and epilepsy lesions of the brain that are resistant to multiple medications. LITT procedures also potentiate immunotherapy and chemotherapy through the transient breakdown of the peritumoral blood tumor barrier, which improves drug delivery to tumors [4] (Table 1).

**Table 1:** Indications and contraindications for laser interstitial thermal therapy (LITT) in neurosurgical procedures [1]

Indications	Contraindications
High-grade Gliomas (grades III-IV) Low-grade Gliomas (grades I-II): deep lesions, recurrent tumors, high risk for craniotomy with systemic disease	Tumors that are large and irregular (chances of damage to normal brain tissue and increased chances of post-procedure cerebral edema and intracranial pressure [ICP])
Selective meningiomas (Primary vs. Recurrent)	Intracranial hemorrhage
Primary or recurrent metastatic tumors	Tumors near major blood vessels (chances of vascular injury, hemorrhage, vasospasm)
Radiation necrosis	Artifacts near the tumors
Refractory seizures resistant to medical management with a known seizure focus	
Primary and metastatic spine tumors	

Two approved LITT systems

**Figure 1:**
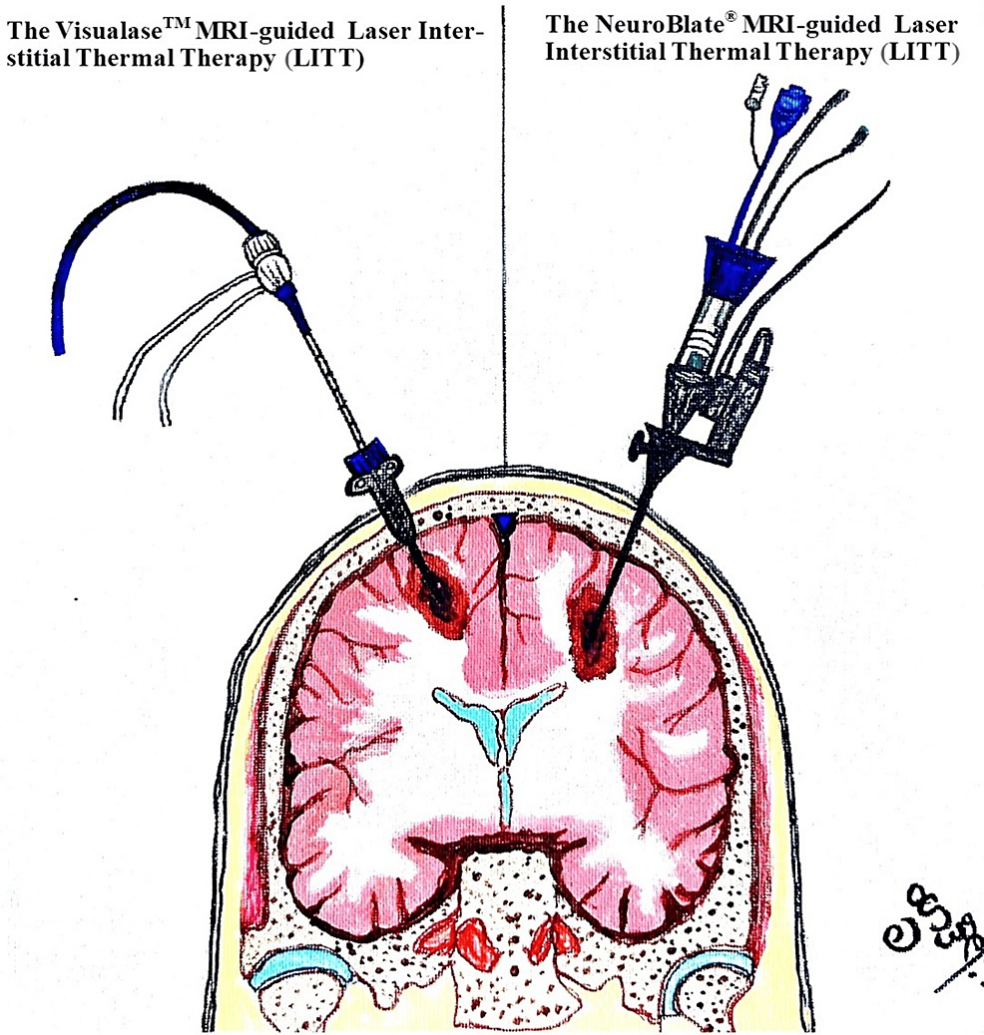
Two types of LITT (laser interstitial thermal therapy) systems for brain tumors: Visualase and NeuroBlate MRI-guided LITT systems. Painting Credit Lakshmi N. Kurnutala M.D., M.Sc.

The Visualase^TM^ (Medtronic Inc., Minneapolis, MN) and the NeuroBlate^®^ (Monteris Medical Inc., Plymouth, MN). There are minimal differences in probe supply energy (Nd:YAG or diode), cooling systems (water, saline, or liquid CO_2_), and magnetic resonance thermography/thermometry (MRT) software (Figure 1). However, the anesthetic management is similar for both types of LITT systems.

LITT procedures for tumors >3 cm in size uses multiple trajectories for the treatment. The temperature varies during LITT, between 42° and 60° C, and the duration of each ablation (30 and 180 seconds) is monitored with color changes in MRT. The adjustment of the probe and temperature maximizes the ablation of the tumor [1].

Patient selection and preoperative evaluation

The appropriate patient selection for the LITT procedure was made by neurosurgery, neurology, and radiation oncology after discussing different treatment options. A careful preoperative assessment includes a complete history, neurological physical exam, past medical and surgical history, airway exam, laboratory results, and imaging studies. Patient medications, including antiepileptic medications and steroids, are reviewed. Patient questions and concerns are addressed during their pre-anesthesia assessment. Radiation necrosis is a severe local tissue reaction that most commonly occurs 3-12 months after the completion of radiation therapy for primary and secondary metastatic brain tumors with a poor prognosis. The LITT procedure combines diagnostic (to assess tumor recurrence vs. radiation necrosis) and cytoreductive surgery. The procedure is unsuitable for patients who present with symptoms of mass effect with raised intracranial pressure [1].

Type of anesthesia and Intraoperative monitoring

The choice may include general anesthesia with an endotracheal tube (volatile agents/total intravenous anesthesia), sedation with local anesthesia, or a combination of both, depending on the patient's condition and the specific requirements of the neurosurgical team. Standard ASA monitors (EKG, blood pressure, end-tidal CO2, oxygen saturation, and temperature), an arterial line, one or two intravenous accesses, and Foley catheters are recommended. Providing adequate depth of anesthesia during the initial phase and maintaining immobility during the LITT procedure is essential [5] (Table 2).

**Table 2:** Anesthetic considerations for the LITT (laser interstitial thermal therapy) procedure [1]

LITT Procedure – Anesthetic Considerations
General anesthesia (GA) or sedation with local anesthesia: but most institutions prefer GA with endotracheal intubation
Local anesthesia infiltration or scalp blocks to reduce perioperative pain and hemodynamic changes
Antiepileptic medications (Levetiracetam 500-100 mg or Fosphenytoin 500-100 mg) to reduce seizure incidence; steroids (Dexamethasone 10 mg) for LITT-related peritumoral cerebral edema
Antiemetic medication to control postoperative nausea and vomiting and reduce the chances of raised ICP (intracranial pressure)

Transport to MRI and safety precautions

After placement of the bolt and probe through a mini craniotomy incision in the operating room under general anesthesia, these patients are transported to an MRI suite for interstitial thermal therapy. Some medical centers have operating rooms equipped with MRI suites, where probe placement and ablation are performed in the same area. During transport, patients are adequately anesthetized and ventilated with an ambu bag with oxygen and standard monitors attached, with particular attention to not disturbing the bolt and probe system. These patients are transferred to the MRI table after ensuring the patient and personnel entering the MRI do not have anything that is MRI-incompatible. In most hospitals, MRI suites are offsite locations and away from the main operating rooms; additional anesthesia personnel, difficult airway equipment that is MRI compatible, emergency medications, and emergency call help are mandatory to improve patient care for these procedures.

Patient positioning in MRI

The LITT procedure is performed in various positions depending on the location of the lesion in the brain. Patients with supratentorial tumors or epilepsy foci are performed in a supine or lateral decubitus position. Patients with infratentorial tumors and spine lesions are performed in the prone position.

LITT and MRI 

During the LITT procedure, the MRI scanner is connected to a computer that runs the software to integrate the images acquired from the scanner in real-time. This integration helps with the real-time position of the probe and the advancement and withdrawal of the probe, measuring the tissue temperature using MRT. MRT helps the surgeon maximize the target lesion ablation, avoiding damage to normal tissue. The Arrhenius equation predicts cell death as a function of tissue temperature and duration of ablation and displays zones of ablation (thermal damage thresholds) [6]. The Arrhenius equation (k=Ae−E_a_/RT) is an expression that provides a relationship between the rate constant (of a chemical reaction), the absolute temperature, and the Ae factor (pre-exponential factor; it can be visualized as the frequency of correctly oriented collisions between reactant particles); RT is average kinetic energy, and E_a_ is active energy. It provides insight into the dependence of reaction rates on the absolute temperature.

Collaboration with neurosurgery and radiology

Effective close communication with the anesthesiology, neurosurgical team, radiology, and ancillary staff is vital in the operating room, during transport, and in MRI. Adequate depth of anesthesia is provided during thermal therapy in the MRI suite with intermittent apnea limited to 100 seconds, or SpO_2_ falls below 94%, whichever is earlier [2]. After completing interstitial thermal therapy, the bolt and probe system are removed, and the patients are transferred to a CT scan to rule out any iatrogenic intracranial hematoma.

Postoperative complications and recovery

Anesthesia providers are crucial in assessing the patient's readiness for extubation, managing postoperative pain, and ensuring a smooth transition to the post-anesthesia care unit (PACU) or the intensive care unit (ICU). Complications related to the LITT procedure are catheter misplacement with damage to the normal brain, TIA (transient ischemic attack), permanent neurological damage, intracranial hemorrhage, seizures, cerebral edema, raised intracranial pressure, and infection [1]. As the size of the brain tumor (>3 cm) increases, the chances of cerebral edema are higher, and there is a potential need for post-procedure decompressive craniotomy [6].

Future advances in LITT

The initial setup cost for the LITT procedure is high; only a few centers across the USA practice these procedures. The current indications are brain tumors, epileptic lesions, and spinal tumors. The primary considerations for the LITT procedure are the heterogeneity of brain tumors, regional blood flow to brain tissue, and MRT, which may not be consistent with heat transfer, causing the potential for normal brain tissue injury. Multimodality imaging, overlying preoperative functional MRI, and three-dimensional planning with MRT reduce the chances of thermal injury to the normal brain with maximal ablation of tumor or epileptic lesions and reduce the chances of recurrence. LITT offers the ablation of inoperable brain tumors as palliative therapy. Further real-world data on the LITT procedure are needed to determine its impact on tumor control, survival, and quality of life [7]. Dr. Levy et al. recently published an article on developing an ERAS (enhanced recovery after surgery) protocol for LITT procedures to improve safety, early recovery, and discharge [8].

## Conclusions

The LITT procedures for brain and spine surgery are evolving; physicians must familiarize themselves with these advances to improve patient care. Based on multiple recent studies, LITT is a minimally invasive procedure that is a safe and reliable approach to deep-seated brain tumors, epilepsy lesions, and radiation necrosis. The anesthesiologist provides safe, effective, patient-centered care throughout the LITT procedure, supporting the neurosurgeon, radiology, and ancillary staff. However, more research is needed to firmly confirm the role of LITT in treating brain and spine tumors.
